# The relationship between eGFR slope and subsequent risk of vascular outcomes and all-cause mortality in type 2 diabetes: the ADVANCE-ON study

**DOI:** 10.1007/s00125-019-4948-4

**Published:** 2019-07-13

**Authors:** Megumi Oshima, Min Jun, Toshiaki Ohkuma, Tadashi Toyama, Takashi Wada, Mark E. Cooper, Samy Hadjadj, Pavel Hamet, Stephen Harrap, Giuseppe Mancia, Michel Marre, Bryan Williams, John Chalmers, Mark Woodward, Vlado Perkovic, on behalf of the ADVANCE Collaborative Group

**Affiliations:** 1 The George Institute for Global Health, University of New South Wales, Sydney, Level 5, 1 King St, Newtown, NSW 2042 Australia; 2 Department of Nephrology and Laboratory Medicine, Kanazawa University, Ishikawa, Japan; 3 Diabetes Domain, Baker IDI Heart and Diabetes Institute, Melbourne, VIC Australia; 4 Department of Endocrinology, Institut du Thorax, Inserm, CNRS, CHU Nantes, Nantes, France; 5 Centre de Recherche, Centre Hospitalier de l’Université de Montréal (CRCHUM), Montréal, QC Canada; 6 Department of Physiology, Royal Melbourne Hospital, University of Melbourne, Melbourne, VIC Australia; 7 Department of Medicine and Surgery, University of Milan-Bicocca, Milan, Italy; 8 Department of Endocrinology, Hôpital Bichat-Claude Bernard, Université Paris, Paris, France; 9 Institute of Cardiovascular Science, University College London and National Institute of Health Research UCL Hospitals Biomedical Research Centre, London, UK; 10 The George Institute for Global Health, University of Oxford, 1st Floor, Hayes House, 75 George Street, Oxford, OX1 2BQ UK; 11 Department of Epidemiology, Johns Hopkins University, Baltimore, MD USA

**Keywords:** Cardiovascular disease, eGFR slope, End-stage kidney disease, Mortality, Surrogate endpoint, Type 2 diabetes

## Abstract

**Aims/hypothesis:**

Some studies have reported that annual change in eGFR (eGFR slope) is associated with the future risk of end-stage kidney disease, cardiovascular disease and death in general or chronic kidney disease cohorts. However, the benefits of using eGFR slopes for prediction of major clinical outcomes in diabetes are unclear.

**Methods:**

We used data from the Action in Diabetes and Vascular Disease: Preterax and Diamicron MR Controlled Evaluation (ADVANCE) trial and the ADVANCE Post-Trial Observational Study (ADVANCE-ON). After excluding the first 4 months during which an acute fall in eGFR was induced by the initiation of an ACE inhibitor and diuretic combination agent, eGFR slopes were estimated by linear mixed models, using three measurements of eGFR at 4, 12 and 24 months after randomisation over 20 months, and categorised according to quartiles. Cox regression models were used to evaluate adjusted HRs for the study’s primary outcome, a composite of major renal events, major macrovascular events and all-cause mortality during the subsequent follow-up from 24 months after randomisation.

**Results:**

A total of 8,879 participants (80%) were included in this cohort. The mean age was 65.6 years (SD 6.3), the mean eGFR was 75 ml min^−1^ (1.73 m)^−2^ (SD 17) and the median urinary albumin/creatinine ratio was 14 μg/mg (interquartile range 7–38). The mean eGFR slope was −0.63 ml min^−1^ (1.73 m)^−2^ year^−1^ (SD 1.75). Over a median follow-up of 7.6 years following the 20-month eGFR slope ascertainment period, 2,221 participants (25%) met the primary outcome. An annual substantial decrease in eGFR (lowest 25%, <−1.63 ml min^−1^ [1.73 m]^−2^ year^−1^) was significantly associated with the subsequent risk of the primary outcome (HR 1.30 [95% CI 1.17, 1.43]) compared with a stable change in eGFR (middle 50%, −1.63 to 0.33). An annual substantial increase in eGFR (highest 25%, >0.33) had no significant association with the risk of the primary outcome (HR 0.96 [95% CI 0.86, 1.07]).

**Conclusions/interpretation:**

Our study supports the utility of eGFR slope in type 2 diabetes as a surrogate endpoint for renal outcomes, as well as a prognostic factor for identifying individuals at high risk of cardiovascular disease and all-cause mortality.

**Trial registry number:**

ClinicalTrials.gov registration no. NCT00145925 and no. NCT00949286

**Electronic supplementary material:**

The online version of this article (10.1007/s00125-019-4948-4) contains peer-reviewed but unedited supplementary material, which is available to authorised users.

## Introduction

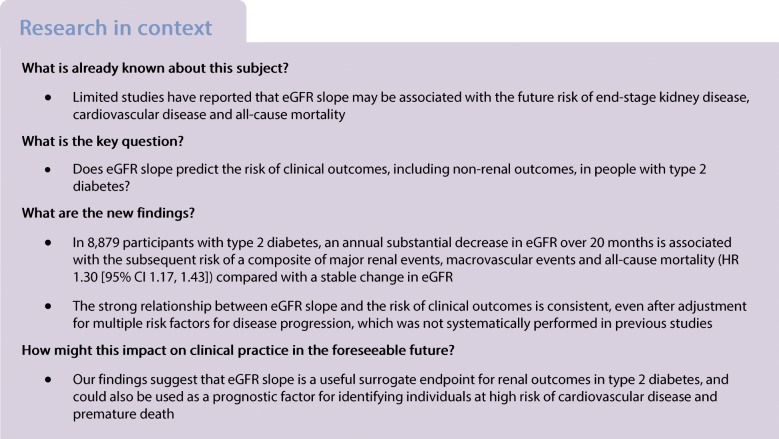



Diabetic kidney disease develops in approximately 40% of individuals with diabetes and can lead to poor outcomes such as end-stage kidney disease (ESKD), cardiovascular disease and premature death [1–4]. It is generally well known that people with diabetes have more rapid decline in kidney function compared with those without diabetes [5–7]. Early recognition of diabetic kidney disease progression is thus critical for the prevention of such adverse long-term outcomes.

In recent years, there has been a growing interest in the assessment of the prognostic utility of short-term changes in eGFR, as well as the therapeutic utility of agents that might ameliorate these changes. Previous meta-analyses have shown significant associations among 30% and 40% decline in eGFR with subsequent risks of ESKD and mortality in individuals with and without diabetes [8, 9]. However, percentage change in eGFR in these studies was calculated using only two measurements, which largely ignores the trajectory of eGFR over time. Accordingly, recent studies have assessed eGFR slope-based approaches using multiple measurements of eGFR to determine the associations between annual change in eGFR and subsequent risk of ESKD [10], cardiovascular disease [11] and all-cause mortality [12–14]. However, such studies have been limited due to the inclusion of participants with advanced chronic kidney disease (CKD) [10, 13, 14] and relatively short periods of follow-up [11, 12].

The objective of our study was thus to examine the prognostic value of eGFR slope in predicting clinical outcomes in individuals with type 2 diabetes, using data from the Action in Diabetes and Vascular disease: Preterax and Diamicron MR Controlled Evaluation (ADVANCE) trial, an RCT in individuals with type 2 diabetes, and its post-trial follow-up (ADVANCE Post-Trial Observational Study [ADVANCE- ON]), which has followed participants for up to 10 years post randomisation.

## Methods

### Study design and population

Our study used data from the ADVANCE and ADVANCE-ON studies. ADVANCE (ClinicalTrials.gov registration no. NCT00145925) was a 2 × 2 factorial RCT evaluating the effects of BP-lowering and intensive blood glucose-lowering treatment on vascular outcomes in individuals with type 2 diabetes. A detailed description of the design has been published previously [15–17]. In brief, a total of 11,140 individuals with type 2 diabetes aged ≥55 years at high risk of cardiovascular events were recruited from 215 centres in 20 countries between June 2001 and March 2003. After a 6-week run-in period on open fixed low-dose perindopril-indapamide (2.0 mg/0.625 mg) and usual glucose-lowering treatment, participants were randomly assigned in a factorial design to the two treatment comparisons: a double blind comparison of the perindopril-indapamide combination (initially 2.0 mg/0.625 mg increasing to 4.0 mg/1.25 mg daily after 3 months) compared with matching placebo; and an open comparison of gliclazide-based intensive therapy (target HbA_1c_ ≤48 mmol/mol [6.5%]) compared with standard therapy for glucose control based on routine guidelines. The median durations of follow-up for the BP- and glucose-lowering trial interventions were 4.4 and 5.0 years, respectively. The ADVANCE-ON study (ClinicalTrials.gov registration no. NCT00949286) was a post-trial follow-up study, comprising 8,494 of the 10,082 surviving participants at the end of the randomised treatment phase [18]. The median total follow-up period (i.e. including both ADVANCE and ADVANCE-ON) was 9.9 years until the final visits which occurred between January 2013 and February 2014. Approvals for the original trial and the post-trial follow-up phase were obtained from the institutional review board of each centre and all participants provided written informed consent.

### Derivation of eGFR slope

**Fig. 1:**
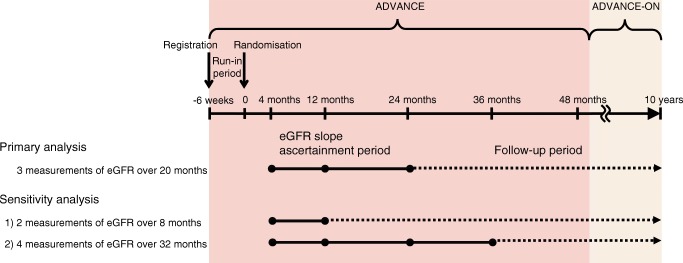
Study design

The current study was restricted to those with three measurements of eGFR within a baseline period (hereinafter referred to as the eGFR slope ascertainment period) (Fig. 1). Participants assigned to BP-lowering medication had an acute fall in eGFR during the first 4 months after randomisation compared with those assigned to placebo. An acute fall in eGFR is generally known to be induced by the initiation of BP-lowering medication that blocks the renin-angiotensin-aldosterone-system (RAAS), including ACE inhibitors (ACEis) or angiotensin-II receptor blockers (ARBs) [19]. To account for this ACEi-induced fall in eGFR, in the primary analysis, the eGFR slope of each included participant was calculated based on three eGFR measurements recorded at 4, 12 and 24 months after randomisation (i.e., over a 20-month eGFR slope ascertainment period).

### Study outcomes and follow-up

The primary outcome for this study was the composite of major renal events (defined as requirement for chronic dialysis or kidney transplantation, or death from renal disease), major macrovascular events (defined as nonfatal and fatal myocardial infarction, nonfatal and fatal stroke or other cardiovascular death) and all-cause mortality. Secondary outcomes included the individual components of the primary outcome. Participants were followed from the end of the eGFR ascertainment period until the first of the study outcomes, death or the end of follow-up (Fig. 1). Study outcomes recorded during the randomised treatment phase were reviewed and validated by an independent endpoint adjudication committee. Outcomes occurring during post-trial follow-up were reported by the study centres using the standardised definitions adopted during the trial, without central adjudication [18].

### Statistical methods

Continuous variables were reported as means with SDs for variables with approximately symmetrical distributions. Results for variables with skewed distributions were presented as median and interquartile range (IQR) and were transformed into natural logarithms before analysis. We summarised baseline characteristics according to quartiles of eGFR slope (<−1.63 ml min^−1^ [1.73 m]^−2^ year^−1^ [lowest 25%; defined as ‘substantial decrease in eGFR’], −1.63 to 0.33 [middle 50%; defined as ‘stable eGFR’] and >0.33 [highest 25%; defined as ‘substantial increase in eGFR’]). Linear trends across categories of eGFR slope were tested by linear regression analysis and logistic regression analysis, as appropriate.

The eGFR slope was estimated using linear mixed models with random intercept. Cox regression models were used to estimate the adjusted HRs and their corresponding 95% CIs for categories of eGFR slope and to compare a substantial decrease in eGFR with stable eGFR and a substantial increase in eGFR with stable eGFR. Cox models were adjusted for covariates including registration values of age, sex, region of residence (Asia or non-Asia), duration of diabetes, log-transformed urine albumin/creatinine ratio (UACR), systolic BP and diastolic BP, a history of macrovascular disease, smoking habits, drinking habits, treated hypertension, HbA_1c_, HDL-cholesterol, LDL-cholesterol, log-transformed triacylglycerol and BMI, 4-month eGFR (at the beginning of the eGFR slope ascertainment period, calculated using the Chronic Kidney Disease Epidemiology Collaboration (CKD-EPI) creatinine equation [20]), and ADVANCE randomised treatment allocation (BP and glucose treatment). A test for linear trend was performed using the category of eGFR slope as a continuous variable in Cox models. An additional quadratic term for eGFR slope was also fitted in Cox models to test for a residual quadratic effect. In addition, restricted cubic splines for eGFR slopes were fitted using no change in eGFR (0 ml min^−1^ [1.73 m]^−2^ year^−1^) as the reference point (knots were placed at −5, −3, −1, 1 and 3 ml min^−1^ [1.73 m]^−2^ year^−1^, as used in previous studies [10, 14]).

We performed the subgroup analyses according to covariates including sex, region of residence, eGFR (<60, 60–89 or ≥90 ml min^−1^ [1.73 m]^−2^) and UACR (<30, 30–300 or >300 μg/mg) at registration and ADVANCE randomised treatment allocation (BP- and glucose-lowering treatment). For sensitivity analyses, we repeated the analysis by using eGFR measurements at the following study points (Fig. 1): (1) 4 and 12 months after randomisation (i.e., over an 8-month eGFR slope ascertainment period); and (2) 4, 12, 24 and 36 months after randomisation (i.e., over a 32-month eGFR slope ascertainment period).

Considering that both 30% and 40% declines in eGFR are sometimes used as surrogate endpoints for CKD progression, sensitivity analyses used percentage change in eGFR (calculated based on two eGFR measurements recorded at 4 and 24 months after randomisation) instead of eGFR slope. We used the Akaike information criterion, the Schwarz’s Bayesian information criterion and c-statistics to assess the discrimination of the Cox models for predicting study outcomes by including eGFR slope or percentage change in eGFR in addition to covariates.

All analyses were conducted using Stata/MP, version 15 (Stata Corporation, College Station, TX, USA). A two-sided *p* value <0.05 was considered statistically significant.

## Results

### Patient characteristics

Of the 11,140 participants in the ADVANCE trial, 8,879 individuals (80%) were included in our final cohort (electronic supplementary material [ESM] Fig. 1). The mean age of the cohort was 65.6 years (SD 6.3), 58% were men, the mean duration of diabetes was 7.8 years at registration (SD 6.3), the mean eGFR was 75 ml min^−1^ (1.73 m)^−2^ (SD 17) and the median UACR was 14 μg/mg (IQR 7–38) (Table 1). Registration characteristics of participants in this cohort were approximately similar to those of the entire trial population (ESM Table 1) [16, 17]. The mean annual change in eGFR was −0.63 ml min^−1^ (1.73 m)^−2^ year^−1^ (SD 1.75) (ESM Fig. 2). Compared with participants with stable eGFR (middle 50%, −1.63 to 0.33 ml min^−1^ [1.73 m]^−2^ year^−1^), those with a substantial decrease in eGFR (lowest 25%, <−1.63) were more likely to be older, a non-smoker, to have a history of macrovascular disease and to have higher levels of albuminuria and lower levels of eGFR. In the multivariable analysis for risk factors associated with eGFR slopes (ESM Table 2), the mean eGFR slope of decline was steeper in individuals with older age, higher levels of UACR and HbA_1c_, and lower level of HDL-cholesterol, while the mean eGFR slope was flatter in individuals with lower eGFR.

**Table 1:** Registration characteristics according to categories of eGFR slopes

Characteristic	Total	eGFR slopes (ml min^-1^ [1.73 m]^-2^ year^-1^)			
		Substantial decrease in eGFR Lowest 25% (<-1.63)	Stable eGFR Middle 50% (-1.63 to 0.33)	Substantial increase in eGFR Highest 25% (>0.33)	p for trend
*N*	8879	2220	4440	2219	
Age (years; mean [SD])	65.6 (6.3)	66.0 (6.4)	65.8 (6.3)	64.6 (6.1)	<0.001
Men (*n* [%])	5108 (58)	1160 (52)	2730 (61)	1218 (55)	0.08
Residence in Asia (*n* [%])	3523 (40)	915 (41)	1590 (36)	1018 (46)	0.002
Duration of diabetes (years; mean [SD])	7.8 (6.3)	8.1 (6.2)	7.6 (6.2)	8.0 (6.5)	0.86
History of macrovascular disease (*n* [%])	2742 (31)	741 (33)	1349 (30)	652 (29)	0.004
Current treated hypertension (*n* [%])	6050 (68)	1580 (72)	2988 (67)	1482 (67)	0.002
Current smoking (*n* [%])	1224 (14)	255 (11)	617 (14)	352 (16)	<0.001
Current alcohol drinking (*n* [%])	2638 (30)	561 (25)	1466 (33)	611 (28)	0.10
UACR (μg/mg; median [IQR])	14 (7–38)	17 (8–48)	14 (7–35)	13 (7–33)	<0.001
eGFR (ml min^−1^ [1.73 m]^−2^; mean [SD])	75 (17)	74 (18)	75 (18)	77 (16)	<0.001
Systolic BP (mmHg; mean [SD])	145 (21)	145 (22)	144 (21)	144 (21)	0.04
Diastolic BP (mmHg; mean [SD])	81 (11)	80 (11)	81 (11)	81 (11)	0.35
HbA_1c_ (mmol/mol; mean [SD])	58 (16)	60 (17)	58 (16)	57 (16)	<0.001
HbA_1c_ (%; mean [SD])	7.5 (1.5)	7.6 (1.6)	7.5 (1.5)	7.4 (1.5)	<0.001
HDL-cholesterol (mmol/l; mean [SD])	1.3 (0.3)	1.2 (0.3)	1.3 (0.3)	1.3 (0.4)	0.002
LDL-cholesterol (mmol/l; mean [SD])	3.1 (1.0)	3.1 (1.0)	3.1 (1.0)	3.2 (1.1)	0.006
Triacylglycerol (mmol/l; median [IQR])	2.0 (1.2–2.3)	1.7 (1.2–2.4)	1.6 (1.2–2.3)	1.6 (1.1–2.3)	0.21
BMI (kg/m^2^; mean [SD])	28.2 (5.2)	28.3 (5.5)	28.3 (5.1)	27.9 (4.9)	0.01
Randomised BP-lowering treatment (*n* [%])	4438 (50)	1180 (53)	2199 (50)	1059 (48)	<0.001
Randomised intensive blood glucose control (*n* [%])	4486 (51)	1151 (52)	2249 (51)	1086 (49)	0.05

Asia includes China, India, Malaysia and the Philippines

### Clinical outcomes during follow-up

**Fig. 2:**
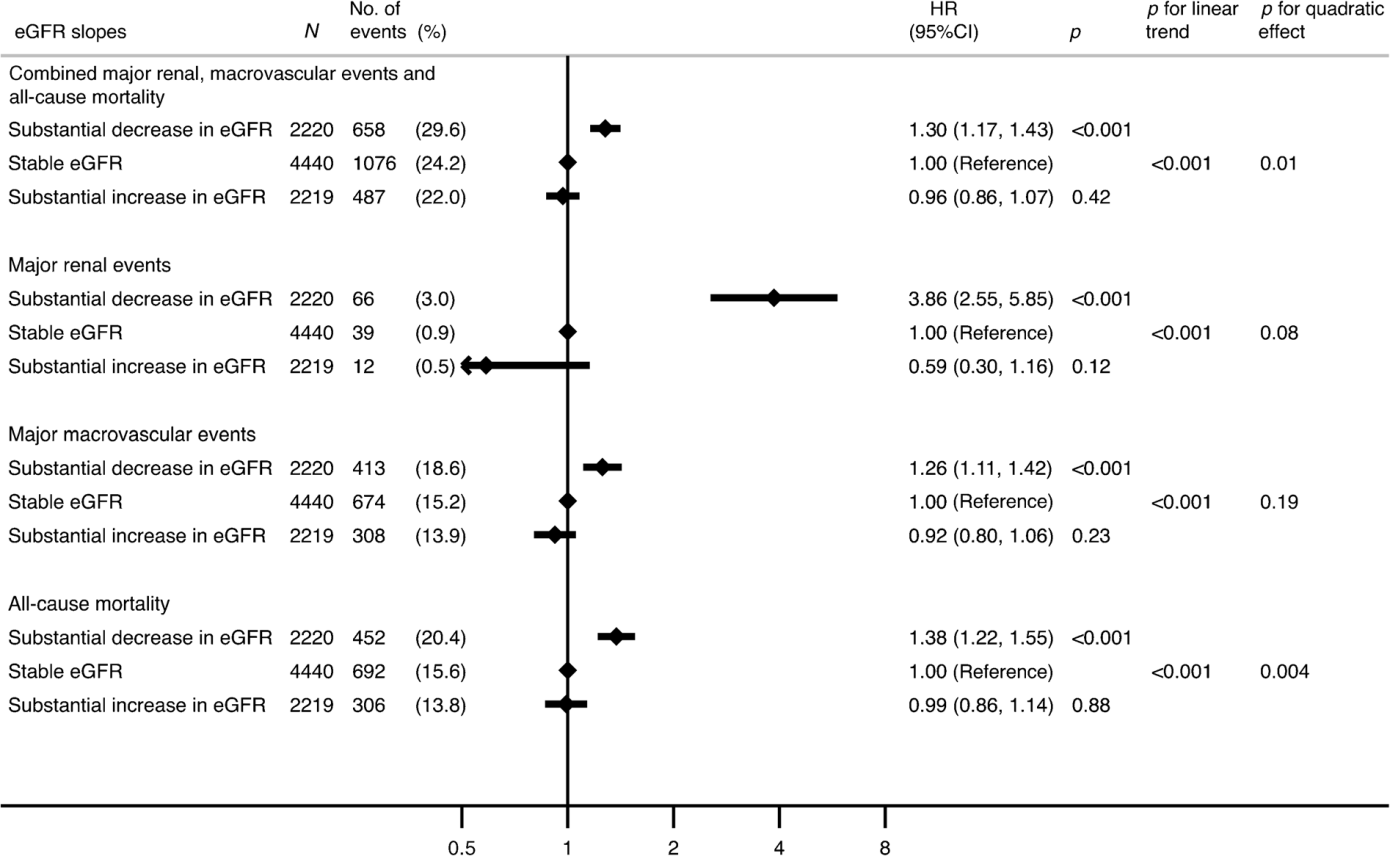
Adjusted HRs for study outcomes according to categories of eGFR slope over the 20-month eGFR slope ascertainment period. Covariates: registration values of age, sex, region of residence, duration of diabetes, log-transformed UACR, systolic BP, diastolic BP, a history of macrovascular disease, smoking, drinking, treated hypertension, HbA_1c_, HDL-cholesterol, LDL-cholesterol, log-transformed triacylglycerol and BMI, 4-month eGFR and randomised treatment allocation (BP and glucose treatment)

**Fig. 3:**
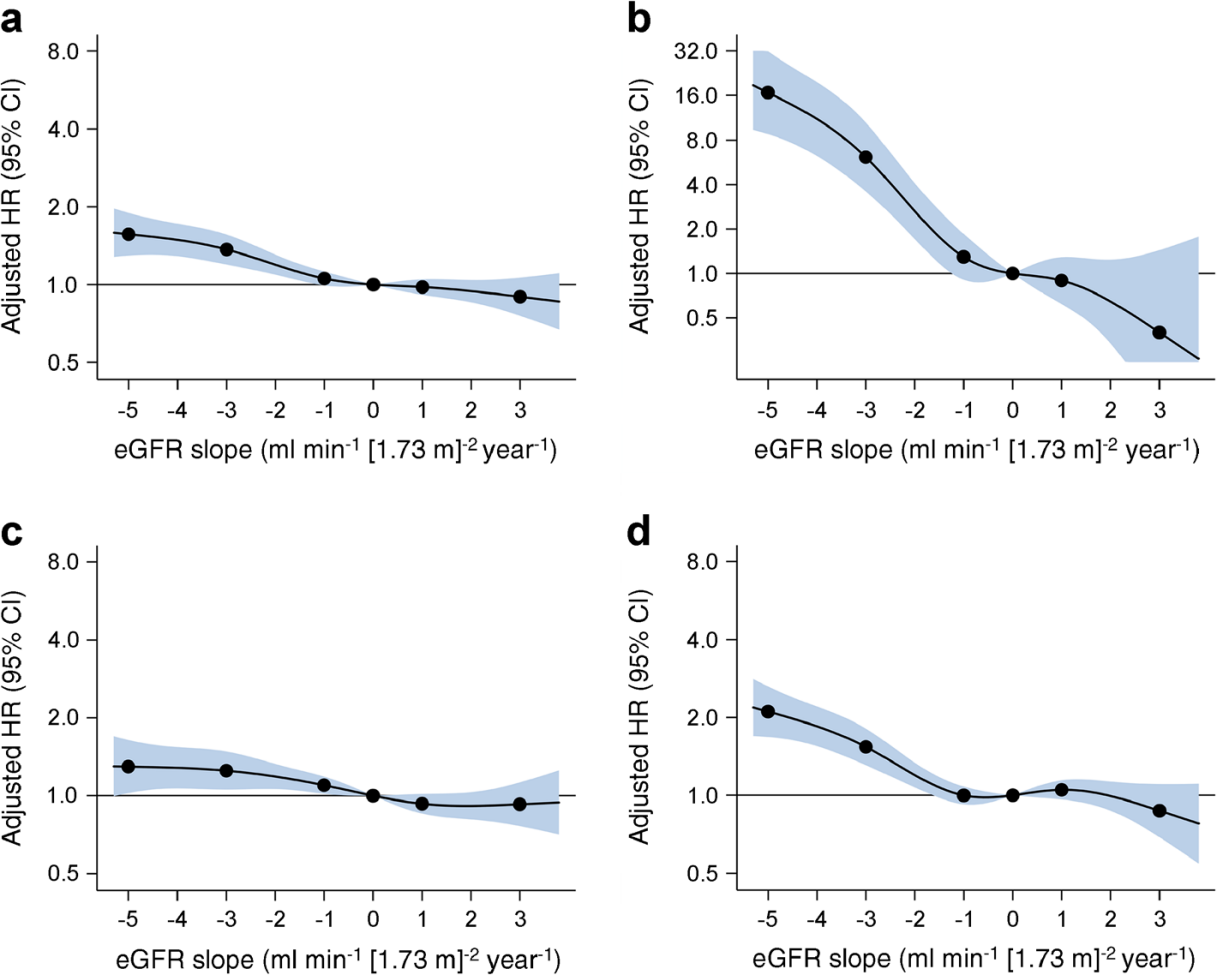
Spline curves showing adjusted HRs and 95% CIs (shaded) for (**a**) combined major renal events, macrovascular events and all-cause mortality (primary outcome), (**b**) major renal events, (**c**) major macrovascular events and (**d**) all-cause mortality, associated with eGFR slopes over the 20-month eGFR slope ascertainment period. Values were trimmed at a slope of <−5.4 and >3.8 ml min^-1^ (1.73 m)^-2^ year^-1^ (each included 1.0% of participants). Knots were placed at -5, -3, -1, 1 and 3 ml min^-1^ (1.73 m)^-2^ year^-1^, using 0 ml min^-1^ (1.73 m)^-2^ year^-1^ as the reference point. Covariates: registration values of age, sex, region of residence, duration of diabetes, log-transformed UACR, systolic BP, diastolic BP, a history of macrovascular disease, smoking, drinking, treated hypertension, HbA_1c_, HDL-cholesterol, LDL-cholesterol, log-transformed triacylglycerol and BMI, 4-month eGFR and randomised treatment allocation (BP and glucose treatment)

Over a median follow-up period of 7.6 years (IQR 3.8–8.7) following the 20-month eGFR slope ascertainment period, 2,221 participants (25%) met the primary composite outcome (117 major renal events [1.3%], 1,395 major macrovascular events [16%] and 1,450 deaths [16%]). Overall, we observed a strong negative linear association between the category of eGFR slope and subsequent risk of the primary composite outcome (*p* for linear trend <0.001; *p* for quadratic effect 0.01; Fig. 2). Compared with stable eGFR, a substantial decrease in eGFR was significantly associated with an increased risk of the primary study outcome (HR 1.30 [95% CI 1.17, 1.43]; *p* < 0.001), whereas a substantial increase in eGFR had no effect (HR 0.96 [95% CI 0.86, 1.07]; *p* < 0.42). We observed similar associations between categories of eGFR slope and the risk of the individual components of the primary composite outcome: increased risk for a substantial decrease in eGFR, but no evidence of a difference in risk for a substantial increase in eGFR, compared with stable eGFR. As shown in Fig. 3, greater annual declines in eGFR were associated with higher risks of study outcomes compared with no change in eGFR (0 ml min^−1^ [1.73 m]^−2^ year^−1^). Among participants with eGFR slopes of −3 ml min^−1^ (1.73 m)^−2^ year^−1^, adjusted HRs were 1.37 (95% CI 1.20, 1.56) for the primary outcome, 6.14 (3.60, 10.49) for major renal events, 1.25 (1.06, 1.48) for major macrovascular events and 1.54 (1.31, 1.81) for all-cause mortality (ESM Table 3). There was also a significant linear association between estimated eGFR slope and the risk of the primary outcome (*p* for linear trend <0.001; *p* for quadratic effect 0.18) (ESM Table 3).

### Subgroup and sensitivity analysis

Overall trends remained unchanged across participant groups defined by sex, region of residence, eGFR and UACR at registration and randomised treatment allocation (BP- and glucose-lowering treatment) (ESM Fig. 3). In sensitivity analyses, the associations were similar when using four measurements of eGFR over the 32-month eGFR slope ascertainment period, but flatter when using two measurements of eGFR over the 8-month eGFR slope ascertainment period, compared with the primary analysis using three measurements over the 20-month eGFR slope ascertainment period (ESM Fig. 4).

Similar associations were observed when assessing percentage change in eGFR (ESM Fig. 5). Compared with the model including percentage change in eGFR in addition to covariates, replacing percentage change in eGFR with eGFR slope did not significantly change the discrimination for predicting clinical outcomes (ESM Table 4).

## Discussion

In a post hoc analysis of 8,879 participants from a large RCT, we observed a strong association between annual change in kidney function and the future risk of major clinical outcomes in type 2 diabetes. We found that an annual substantial decrease in eGFR over 20 months exhibited a statistically significant association with increased risks of major renal events, major macrovascular events and all-cause mortality, independent of baseline kidney function, albuminuria and other covariates, consistently observed across various patient subgroups of covariates. Although an annual substantial increase in eGFR did not significantly predict the risk of these outcomes, our results from multiple analyses supported the utility of eGFR slopes for predicting the subsequent vascular outcomes and all-cause death in type 2 diabetes.

A limited number of studies have shown the relationship between eGFR slopes and the subsequent risk of ESKD [10, 21], cardiovascular disease [11, 22] and all-cause mortality [12–14]. Results from a meta-analysis of 13 CKD cohorts showed that an eGFR slope of −3 vs 0 ml min^−1^ (1.73 m)^−2^ year^−1^ over 3 years was associated with the risk of ESKD (HR 1.73 [95% CI 1.50, 2.00]) after adjusting for last measurement of eGFR [10]. In a cohort of 529,312 adults in the Alberta Kidney Disease Network, an eGFR slope of −4 ml min^−1^ (1.73 m)^−2^ year^−1^ was associated with 74%, 16% and 21% higher risks of congestive heart failure, acute myocardial infarction and stroke, respectively, compared with no change in eGFR [11]. In a cohort of French individuals with type 2 diabetes, annual eGFR decline over 6.3 years of follow-up was greater in individuals with major cardiovascular events compared with those without (−3.0 vs −1.7 ml min^−1^ [1.73 m]^−2^ year^−1^) [22]. Furthermore, a meta-analysis of 12 CKD cohorts within the CKD Prognosis Consortium showed that an eGFR slope of −6 vs 0 ml min^−1^ (1.73 m)^−2^ year^−1^ over 3 years was associated with an adjusted HR for all-cause mortality of 1.25 (95% CI 1.09, 1.44) after adjusting for last measurement of eGFR [14]. However, these results were mostly based on general or CKD populations. Therefore, the current study was notable for evaluating the composite outcome of major renal and macrovascular events and all-cause mortality in a large population with type 2 diabetes after adjusting for important risk factors of kidney disease progression.

Various mechanisms have been suggested to explain why declining kidney function is associated with increased risk of cardiovascular disease and all-cause mortality as well as renal outcome. Decrease in eGFR may exacerbate cardiovascular risk factors such as BP and lipids [23]. Other possible factors which were not measured in our cohort include activation of the RAAS, endothelial dysfunction, inflammation and oxidative stress [24, 25]. On the other hand, these risk factors and progression of cardiovascular disease certainly accelerate the progression of CKD [26]. In addition, worsening kidney function may cause decreased appetite, decreased physical function and overall frailty, and indirectly result in higher mortality risk [14].

There has been increasing interest in the utility of eGFR slope as a surrogate endpoint for predicting subsequent ESKD in clinical trials, but a clear definition of the magnitude of eGFR slope as a surrogate endpoint has not been established. The 2012 Kidney Disease: Improving Global Outcomes (KDIGO) guideline defined rapid eGFR decline as a sustained decline in eGFR of greater than −5 ml min^−1^ (1.73 m)^−2^ year^−1^ [27], which was supported by other studies [10, 21]. A number of studies already used eGFR slope greater than −5 ml min^−1^ (1.73 m)^−2^ year^−1^ as a substitute for kidney outcome, which suggested the potential of eGFR slope of −5 ml min^−1^ (1.73 m)^−2^ year^−1^ as a surrogate endpoint. However, in the present study (approximately 80% had early-stage diabetic kidney disease at baseline with eGFR ≥60 ml min^−1^ [1.73 m]^−2^ and UACR <300 μg/mg), only 1.4% of participants developed eGFR decline greater than −5 ml min^−1^ (1.73 m)^−2^ year^−1^. Instead, more than fivefold participants developed eGFR slopes of −3 vs −5 ml min^−1^ (1.73 m)^−2^ year^−1^ and their risks of major renal events were weaker but still robustly increased. Therefore, the potential of using eGFR slopes less than −5 ml min^−1^ (1.73 m)^−2^ year^−1^ may be a future subject to be assessed for seeking more practical surrogate endpoints in people with type 2 diabetes.

The standard duration for estimating eGFR slopes is also unknown. We excluded the first 4 months after randomisation from the eGFR slope ascertainment period, in order to remove an acute pharmacological effect of ACEi on eGFR. RAAS blockers, including ACEis and ARBs, are largely known to prevent the onset and progression of diabetic kidney disease and improve survival rate in people with diabetes [28–30]. During the initiation of RAAS blockers, there may be an acute fall in eGFR [19, 31], because RAAS blockers inhibit angiotensin 2-mediated renal vasoconstriction which in turn causes a reduction in intraglomerular pressure and filtration fraction. Thus, in people starting RAAS blockers, changes in eGFR over time should be evaluated separately during the initial months when an acute fall in eGFR is observed, and during subsequent periods until the end of follow-up.

Currently, both 30% and 40% declines in eGFR are widely accepted as surrogate endpoints for CKD progression, based on a series of meta-analyses and simulations [8, 9]. In this study, similar associations were observed when using percentage change in eGFR and eGFR slope, and the discrimination statistics in the model including eGFR slope were similar to those using percentage change in eGFR for predicting major clinical outcomes. This may be consistent with a previous report [32]. The eGFR slope can potentially reflect the course of changes in kidney function more accurately than percentage change in eGFR, because the slope takes into account all of the available eGFR measurements of an individual over time. Our study indicated that evaluating eGFR slope in an individual might be a promising alternative to percentage change in eGFR for predicting the progression of diabetic kidney disease.

Recently, a number of clinical trials have assessed the effects of sodium glucose cotransporter 2 inhibitors in people with type 2 diabetes. Among the trials with similar baseline renal characteristics to our cohort, the Canagliflozin Cardiovascular Assessment Study (CANVAS) Program (mean eGFR 77 ml min^−1^ [1.73 m]^−2^ [SD 21]) reported that participants allocated to the placebo group had a mean annual long-term decline in eGFR of −0.9 ml min^−1^ (1.73 m)^−2^ year^−1^ [33]. The Empagliflozin Cardiovascular Outcome Event Trial in Type 2 Diabetes Mellitus Patients–Removing Excess Glucose (EMPA-REG OUTCOME) trial (mean eGFR 74 ml min^−1^ [1.73 m]^−2^ [22]) observed a mean annual change in eGFR of −1.46 ml min^−1^ (1.73 m)^−2^ year^−1^ in the placebo group [34]. Our cohort included a lower-risk population showing slower decline in eGFR compared with these trials.

The strengths of our study include the large number and diverse groups of participants, the long duration of follow-up, the sequential measurements of eGFR during the ADVANCE trial and the ability to adjust for multiple important risk factors. Also, we used linear mixed models, which are more robust than ordinary linear regression models, to estimate eGFR slopes and assess changes over time in participants with varying intervals between measurements [35]. However, our study has several limitations. First, as our study cohort was derived from a randomised trial, the results may limit generalisability to broader populations. Second, only 84% of the participants alive at the end of the ADVANCE trial were enrolled in the post-trial follow-up (ADVANCE-ON trial). However, baseline characteristics of those included in the ADVANCE-ON trial were similar to those of the entire trial population [18]. Third, we used eGFR instead of a directly measured GFR to calculate GFR slopes, which may lead to some misclassification of true course of change in kidney function [36, 37]. Finally, our models to estimate eGFR slopes did not consider non-linear and time-varying patterns of eGFR decline. However, a previous study showed that slopes were linear for 83% of individuals with diabetes and normal kidney function [38]. Also, estimating eGFR slope may be subject to measurement error including regression to the mean [39].

In conclusion, an annual substantial decrease in eGFR over 20 months was strongly associated with the future risk of renal and cardiovascular events and all-cause mortality in type 2 diabetes, supporting the potential for using eGFR slope as a predictor for major clinical outcomes. The present analysis suggests that monitoring eGFR over time is beneficial to identifying individuals with diabetes at high risk of vascular outcomes and all-cause death, requiring close monitoring for early initiation of appropriate preventive and therapeutic strategies.

## Electronic supplementary material

ESM 1 (PDF 1149 kb)

## Data Availability

The datasets generated and/or analysed during the current study are available from the corresponding author on reasonable request.

## Abbreviations

ACEi: ACE inhibitor
ADVANCE: The Action in Diabetes and Vascular disease: Preterax and Diamicron MR Controlled Evaluation
ADVANCE-ON: ADVANCE Post-Trial Observational Study
ARB: Angiotensin-II receptor blocker
CKD: Chronic kidney disease
ESKD: End-stage kidney disease
IQR: Interquartile range
RAAS: Renin-angiotensin-aldosterone-system
UACR: Urine albumin/creatinine ratio
