# Supplementary material for: The relationship between eGFR slope and subsequent risk of vascular outcomes and all-cause mortality in type 2 diabetes: the ADVANCE-ON study
Source: Diabetologia. 2019 Jul 13;62(11):1988–97. doi: 10.1007/s00125-019-4948-4 (PMC6805825; doi:10.1007/s00125-019-4948-4)
Supplement: Supplementary file 1 — (PDF 1149 kb) [file 125_2019_4948_MOESM1_ESM.pdf]

ESM Table 1 Registration characteristics in this cohort and entire trial population in ADVANCE study.

**Characteristic**	**Entire trial population in ADVANCE study (n=11,140)**	**Included participants in this cohort (n=8,879)**
Age (years; mean [SD])	65.8 (6.4)	65.6 (6.3)
Men (n [%])	6405 (58)	5108 (58)
Residence in Asia (n [%])	4136 (37)	3523 (40)
Duration of diabetes (years; mean [SD])	7.9 (6.4)	7.8 (6.3)
History of macrovascular disease (n [%])	3590 (32)	2742 (31)
Current treated hypertension (n [%])	7655 (69)	6050 (68)
Current smoking (n [%])	1550 (14)	1224 (14)
Current alcohol drinking (n [%])	3396 (30)	2638 (30)
UACR (μg/mg; median [IQR])	15 (7–40)	14 (7–38)
eGFR (ml min^{-1} [1.73 m]$^{-2}$; mean [SD])	74 (18)	75 (17)
Systolic BP (mmHg; mean [SD])	145 (22)	145 (21)
Diastolic BP (mmHg; mean [SD])	81 (11)	81 (11)
HbA_{1c} (mmol/mol; mean [SD])	59 (18)	58 (16)
HbA_{1c} (%; mean [SD])	7.5 (1.6)	7.5 (1.5)
HDL cholesterol (mmol/L; mean [SD])	1.3 (0.4)	1.3 (0.3)
LDL cholesterol (mmol/L; mean [SD])	3.1 (1.0)	3.1 (1.0)
Triacylglycerol (mmol/L; median [IQR])	1.6 (1.2–2.3)	2.0 (1.2–2.3)
BMI (kg/m^2; mean [SD])	28.3 (5.2)	28.2 (5.2)
Randomised BP lowering treatment (n [%])	5569 (50)	4438 (50)
Randomised intensive blood glucose control (n [%])	5571 (50)	4486 (51)

Asia includes China, India, Malaysia and the Philippines.

ESM Table 2 Multivariable analyses of risk factors associated with eGFR slopes over the 20-month eGFR slope ascertainment period according to subgroup at registration

**Risk factor**		**N**	**eGFR slopes ($ml\ min^{-1}\ [1.73\ m]^{-2}\ year^{-1}$; mean [95% CI])**	**Difference (95% CI)**	**p**	**p for trend**
Age (years)	55–64	3690	-0.10 (-0.38, 0.18)	Reference		<0.001
	65–74	4459	-0.94 (-1.19, -0.68)	-0.83 (-1.22, -0.45)	<0.001	
	≥75	730	-1.53 (-2.16, -0.91)	-1.43 (-2.13, -0.73)	<0.001	
Sex	Men	5108	-0.60 (-0.84, -0.37)	Reference		
	Women	3771	-0.68 (-0.96, -0.41)	-0.08 (-0.45, 0.29)	0.67	
History of macrovascular disease	No	6137	-0.52 (-0.73, -0.31)	Reference		
	Yes	2742	-0.90 (-1.22, -0.58)	-0.38 (-0.77, 0.00)	0.05	
UACR (μg/mg)	<30	6249	-0.41 (-0.62, -0.20)	Reference	0.05	<0.001
	30–300	2318	-0.93 (-1.28, -0.59)	-0.52 (-0.93, -0.12)	0.01	
	>300	312	-3.09 (-4.04, -2.15)	-2.70 (-3.67, -1.74)	<0.001	
eGFR ($ml\ min^{-1}\ [1.73\ m]^{-2}$)	≥90	2037	-2.20 (-2.58, -1.82)	Reference		<0.001
	60–89	5010	-0.43 (-0.66, -0.19)	1.77 (1.32, 2.22)	<0.001	
	<60	1832	0.53 (0.13, 0.92)	2.73 (2.16, 3.29)	<0.001	
Systolic BP (mmHg)	<140	3794	-0.55 (-0.82, -0.28)	Reference		
	≥140	5086	-0.70 (-0.94, -0.47)	-0.15 (-0.51, 0.21)	0.40	
HbA_{1c} (mmol/mol [%])	<53 (< 7.0)	3778	-0.11 (-0.37, 0.16)	Reference		<0.001
	53-63 (7.0–7.9)	2509	-0.76 (-1.09, -0.44)	-0.66 (-1.08, -0.23)	0.002	
	≥64 (≥ 8.0)	2425	-1.34 (-1.67, -1.00)	-1.23 (-1.66, -0.80)	<0.001	
HDL cholesterol (mmol/L)	≥1.0	7096	-0.54 (-0.73, -0.34)	Reference		
	<1.0	1783	-1.04 (-1.44, -0.64)	-0.50 (-0.95, -0.05)	0.03	
LDL cholesterol (mmol/L)	<2.6	3025	-0.72 (-1.03, -0.42)	Reference		
	≥2.6	5854	-0.59 (-0.81, -0.37)	0.12 (-0.26, 0.50)	0.53	
BMI (kg/m^2)	<30	6137	-0.58 (-0.79, -0.37)	Reference		
	≥30	2742	-0.76 (-1.08, -0.45)	-0.18 (-0.57, 0.20)	0.35	

ESM Table 3 Adjusted HRs and 95% CIs for study outcomes associated with eGFR slopes over the 20-month eGFR slope ascertainment period

**Clinical outcome**	**eGFR slopes (ml min^{-1} [1.73 m]$^{-2}$ year^{-1})**							**p for linear trend**	**p for quadratic effect**
	**-5**	**-4**	**-3**	**-2**	**-1**	**0**	**1**		
Combined major renal events, macrovascular events and all-cause mortality	1.57 (1.30, 1.90)	1.49 (1.29, 1.72)	1.37 (1.20, 1.56)	1.19 (1.09, 1.31)	1.06 (0.99, 1.12)	1.00 (Reference)	0.98 (0.91, 1.05)	<0.001	0.18
Major renal events	16.70 (8.57, 30.15)	11.09 (6.26, 19.65)	6.14 (3.60, 10.49)	2.65 (1.71, 4.10)	1.30 (0.91, 1.85)	1.00 (Reference)	0.90 (0.62, 1.29)	<0.001	0.48
Major macrovascular events	1.30 (1.02, 1.64)	1.28 (1.07, 1.54)	1.25 (1.06, 1.48)	1.19 (1.06, 1.33)	1.10 (1.02, 1.20)	1.00 (Reference)	0.93 (0.85, 1.02)	0.001	0.80
All-cause mortality	2.11 (1.68, 2.64)	1.85 (1.55, 2.21)	1.54 (1.31, 1.81)	1.20 (1.07, 1.34)	1.00 (0.92, 1.08)	1.00 (Reference)	1.05 (0.96, 1.15)	<0.001	0.005

Covariates: registration values of age, sex, region of residence, duration of diabetes, log-transformed UACR, systolic BP, diastolic BP, a history of macrovascular disease, smoking, drinking, treated hypertension, HbA_{1c}, HDL-cholesterol, LDL-cholesterol, log-transformed triacylglycerol and BMI, 4-month eGFR and randomised treatment allocation (BP and glucose treatment)

ESM Table 4 Discrimination statistics for predicting study outcomes in the models including eGFR slope or percent change in eGFR in addition to covariates

Clinical outcome	Model	AIC	BIC	c-statistic (95% CI)	p
Combined major renal events, macrovascular events and all-cause mortality	eGFR slope	30909.68	31051.26	0.6636 (0.6516, 0.6755)	0.51
	Percent change in eGFR	30911.11	31052.69	0.6633 (0.6514, 0.6752)	
Major renal events	eGFR slope	1401.83	1543.41	0.8679 (0.8322, 0.9035)	0.22
	Percent change in eGFR	1394.66	1536.24	0.8721 (0.8385, 0.9058)	
Major macrovascular events	eGFR slope	19720.89	19862.47	0.6594 (0.6443, 0.6744)	0.41
	Percent change in eGFR	19723.66	19865.25	0.6590 (0.6439, 0.6741)	
All-cause mortality	eGFR slope	19715.13	19856.71	0.7132 (0.6993, 0.7271)	0.93
	Percent change in eGFR	19715.98	19857.56	0.7132 (0.6993, 0.7270)	

Covariates: registration values of age, sex, region of residence, duration of diabetes, log-transformed UACR, systolic BP, diastolic BP, a history of macrovascular disease, smoking, drinking, treated hypertension, HbA_{1c}, HDL-cholesterol, LDL-cholesterol, log-transformed triacylglycerol and BMI, 4-month eGFR and randomised treatment allocation (BP and glucose treatment) AIC, Akaike's information criterion; BIC, Schwarz's Bayesian information criterion.

ESM Fig. 1 The identification of study cohort.

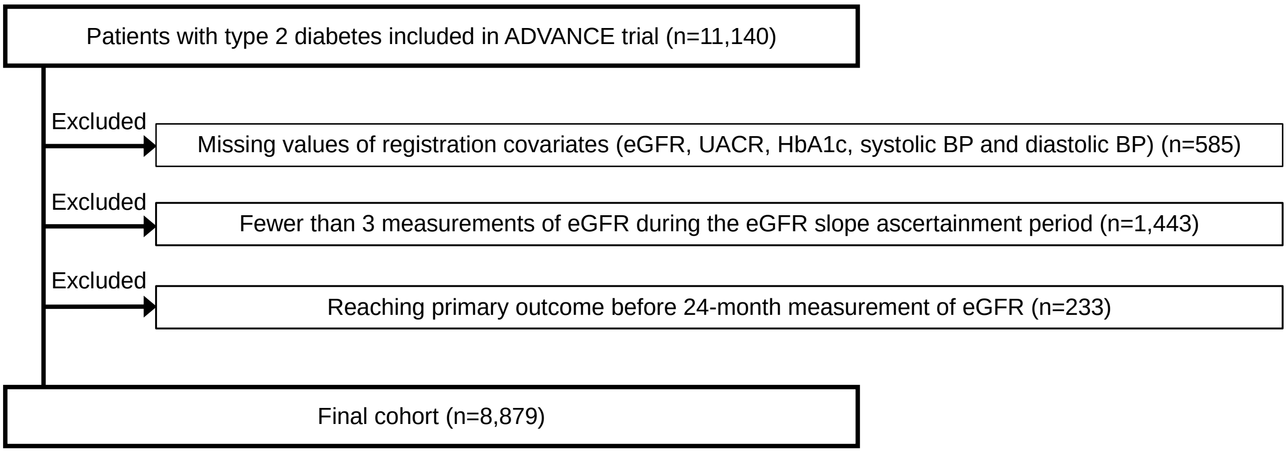

ESM Fig. 2 Distribution of eGFR slopes over the 20-month eGFR slope ascertainment period

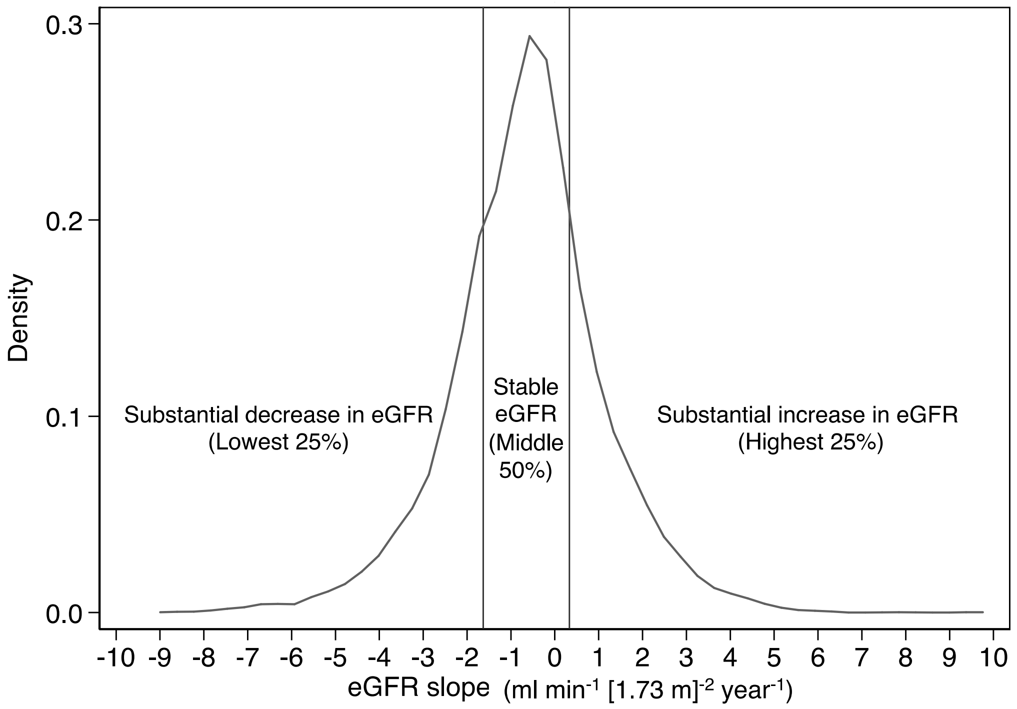

ESM Fig. 3 Adjusted HRs for the composite of major renal events, macrovascular events and all-cause mortality according to categories of eGFR slopes over the 20-month eGFR slope ascertainment period by subgroup at registration

Subgroup	eGFR slope	No. of total	No. of events (%)	Hazard ratio (95%CI)	P for interaction
Sex					
Men	Substantial decrease in eGFR	1277	426 (33.4)	1.27 (1.13, 1.45)	
	Stable eGFR	2554	696 (27.3)	1.00 (Reference)	
	Substantial increase in eGFR	1277	318 (24.9)	0.94 (0.82, 1.08)	0.93
Women	Substantial decrease in eGFR	943	230 (24.4)	1.22 (1.03, 1.45)	
	Stable eGFR	1886	379 (20.1)	1.00 (Reference)	
	Substantial increase in eGFR	942	172 (18.3)	0.88 (0.74, 1.06)	
Region of residence					
Asia	Substantial decrease in eGFR	914	266 (29.1)	1.22 (1.05, 1.43)	
	Stable eGFR	1828	474 (25.9)	1.00 (Reference)	
	Substantial increase in eGFR	913	209 (22.9)	0.88 (0.74, 1.03)	0.49
Others	Substantial decrease in eGFR	1306	384 (29.4)	1.26 (1.10, 1.43)	
	Stable eGFR	2612	617 (23.6)	1.00 (Reference)	
	Substantial increase in eGFR	1306	271 (20.8)	0.99 (0.85, 1.14)	
eGFR (ml min^{-1} [1.73 m]$^{-2}$ year^{-1})					
≥90	Substantial decrease in eGFR	510	107 (21.0)	1.01 (0.79, 1.29)	
	Stable eGFR	1018	208 (20.4)	1.00 (Reference)	
	Substantial increase in eGFR	509	92 (18.1)	0.94 (0.73, 1.22)	
60-89	Substantial decrease in eGFR	1253	341 (27.2)	1.13 (0.99, 1.30)	
	Stable eGFR	2505	611 (24.4)	1.00 (Reference)	0.06
	Substantial increase in eGFR	1252	259 (20.7)	0.87 (0.75, 1.01)	
<60	Substantial decrease in eGFR	458	193 (42.1)	1.28 (1.05, 1.55)	
	Stable eGFR	916	288 (31.4)	1.00 (Reference)	
	Substantial increase in eGFR	458	122 (26.6)	1.02 (0.81, 1.27)	
UACR (µg/mg)					
<30	Substantial decrease in eGFR	1563	390 (25.0)	1.18 (1.04, 1.35)	
	Stable eGFR	3124	687 (22.0)	1.00 (Reference)	
	Substantial increase in eGFR	1562	304 (19.5)	0.90 (0.79, 1.04)	
30–300	Substantial decrease in eGFR	580	211 (36.4)	1.48 (1.24, 1.77)	
	Stable eGFR	1159	347 (29.9)	1.00 (Reference)	0.34
	Substantial increase in eGFR	579	160 (27.6)	1.02 (0.84, 1.23)	
>300	Substantial decrease in eGFR	78	39 (50.0)	1.39 (0.86, 2.24)	
	Stable eGFR	156	56 (35.9)	1.00 (Reference)	
	Substantial increase in eGFR	78	27 (34.6)	1.07 (0.65, 1.76)	
Randomised treatment (Blood pressure/Glucose)					
Placebo/Standard	Substantial decrease in eGFR	551	157 (28.5)	1.16 (0.95, 1.41)	
	Stable eGFR	1102	280 (25.4)	1.00 (Reference)	
	Substantial increase in eGFR	550	118 (21.5)	0.91 (0.73, 1.13)	
Placebo/Intensive	Substantial decrease in eGFR	560	190 (33.9)	1.51 (1.25, 1.82)	
	Stable eGFR	1119	271 (24.2)	1.00 (Reference)	
	Substantial increase in eGFR	559	119 (21.3)	0.86 (0.69, 1.07)	0.30
BP treatment/Standard	Substantial decrease in eGFR	548	146 (26.6)	1.27 (1.03, 1.57)	
	Stable eGFR	1095	261 (23.8)	1.00 (Reference)	
	Substantial increase in eGFR	547	119 (21.8)	1.00 (0.80, 1.25)	
BP treatment/Intensive	Substantial decrease in eGFR	562	159 (28.3)	1.14 (0.93, 1.40)	
	Stable eGFR	1124	273 (24.3)	1.00 (Reference)	
	Substantial increase in eGFR	562	128 (22.8)	0.99 (0.80, 1.23)	

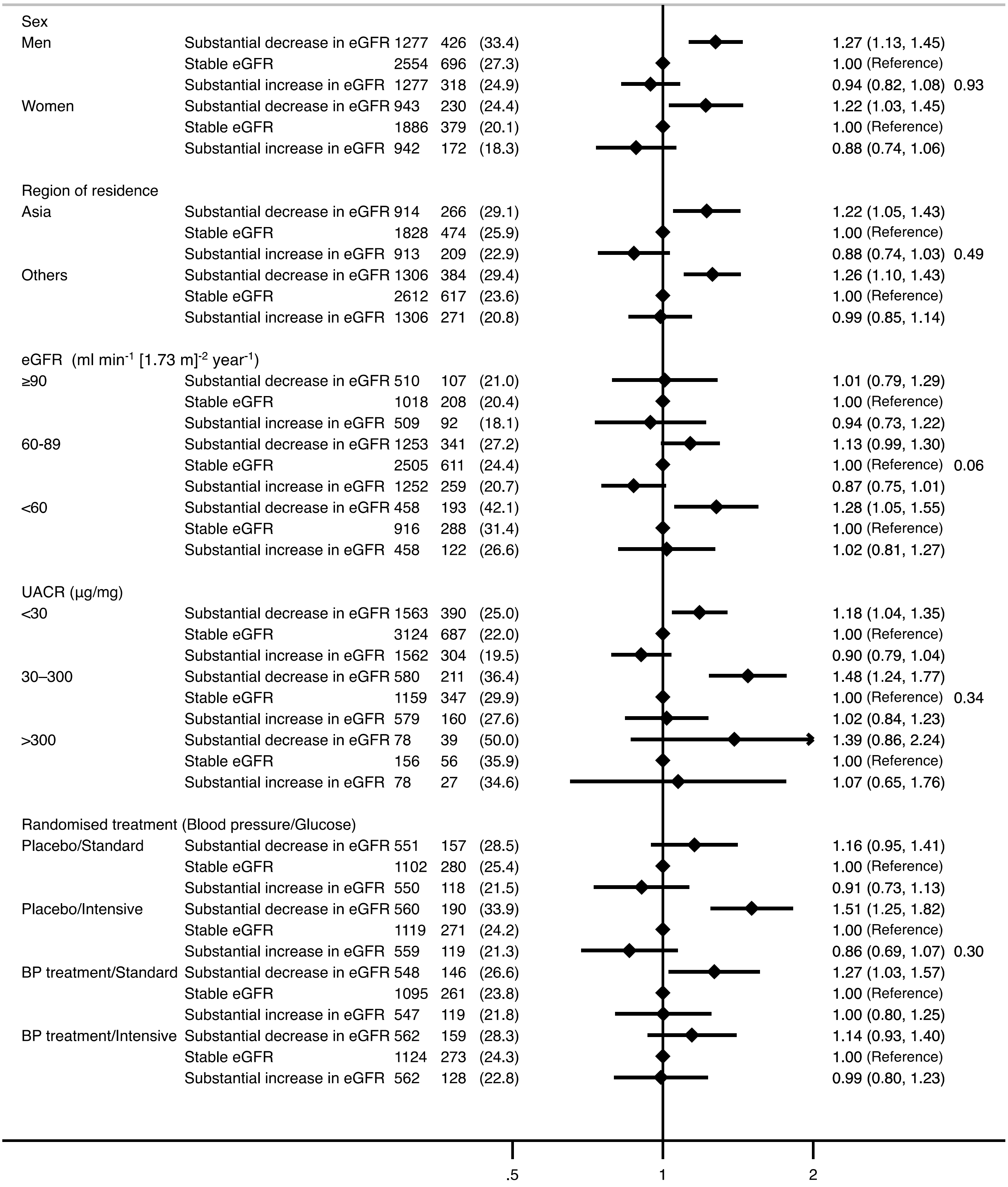

Covariates: registration values of age, sex, region of residence, duration of diabetes, log-transformed UACR, systolic BP, diastolic BP, a history of macrovascular disease, smoking, drinking, treated hypertension, HbA_{1c}, HDL-cholesterol, LDL-cholesterol, log-transformed triacylglycerol and BMI, 4-month eGFR and randomised treatment allocation (BP and glucose treatment)

ESM Fig. 4 Sensitivity analysis: Spline curves showing adjusted HRs and 95% CIs (shaded) for study outcomes associated with eGFR slopes over the 8-, 20- or 32-month eGFR slope ascertainment period

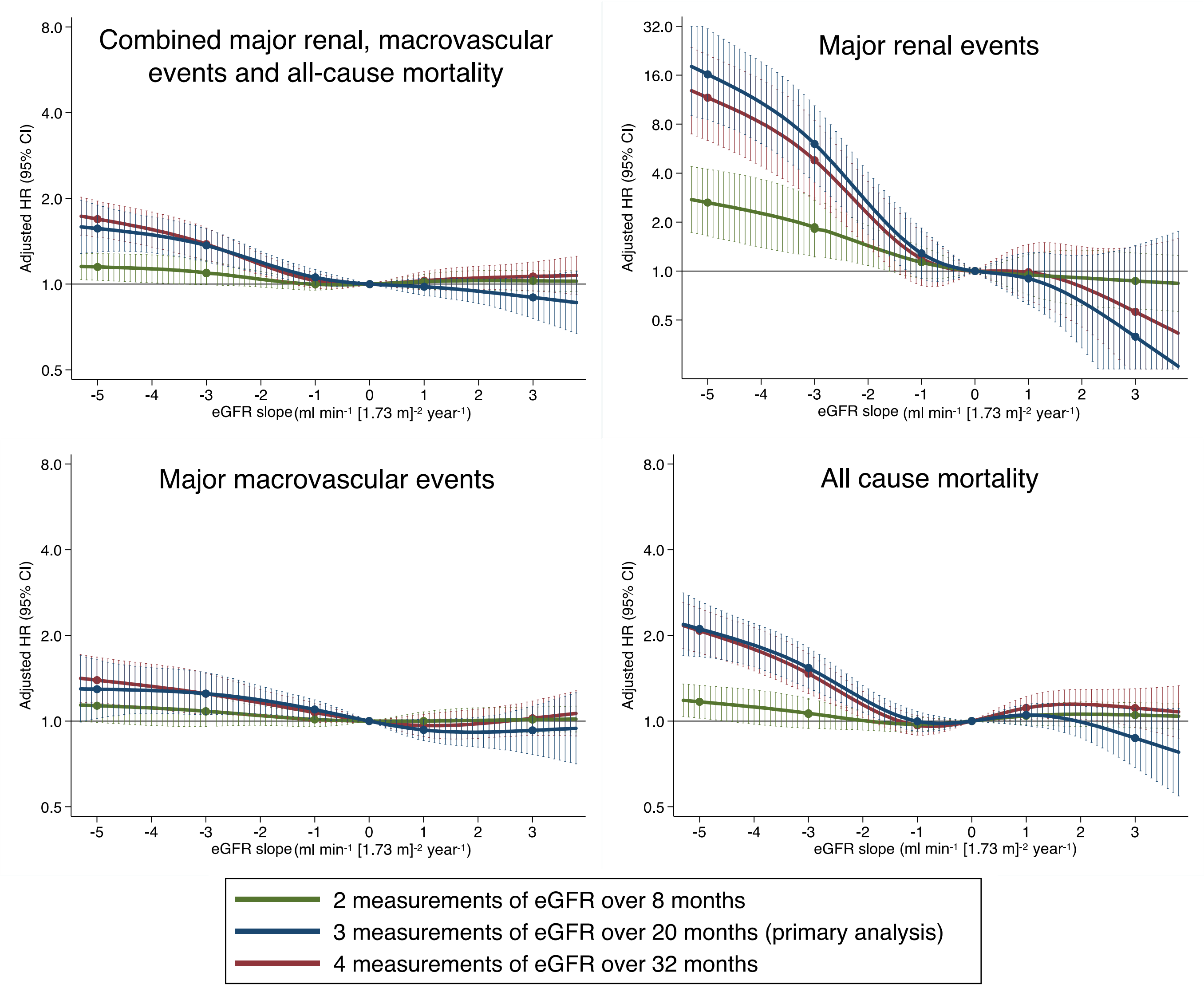

Values were trimmed at a slope of <-5.4 and >3.8 ml min^{-1} (1.73 m)$^{-2}$ year^{-1}. Knots were placed at -5, -3, -1, 1 and 3 ml min^{-1} (1.73 m)$^{-2}$ year^{-1}, using 0 ml min^{-1} (1.73 m)$^{-2}$ year^{-1} as the reference point. Covariates: registration values of age, sex, region of residence, duration of diabetes, log-transformed UACR, systolic BP, diastolic BP, a history of macrovascular disease, smoking, drinking, treated hypertension, HbA_{1c}, HDL-cholesterol, LDL-cholesterol, log-transformed triacylglycerol and BMI, 4-month eGFR and randomised treatment allocation (BP and glucose treatment)

ESM Fig. 5 Spline curves showing adjusted HRs and 95% CIs (shaded) for study outcomes associated with percent change in eGFR over 20 months

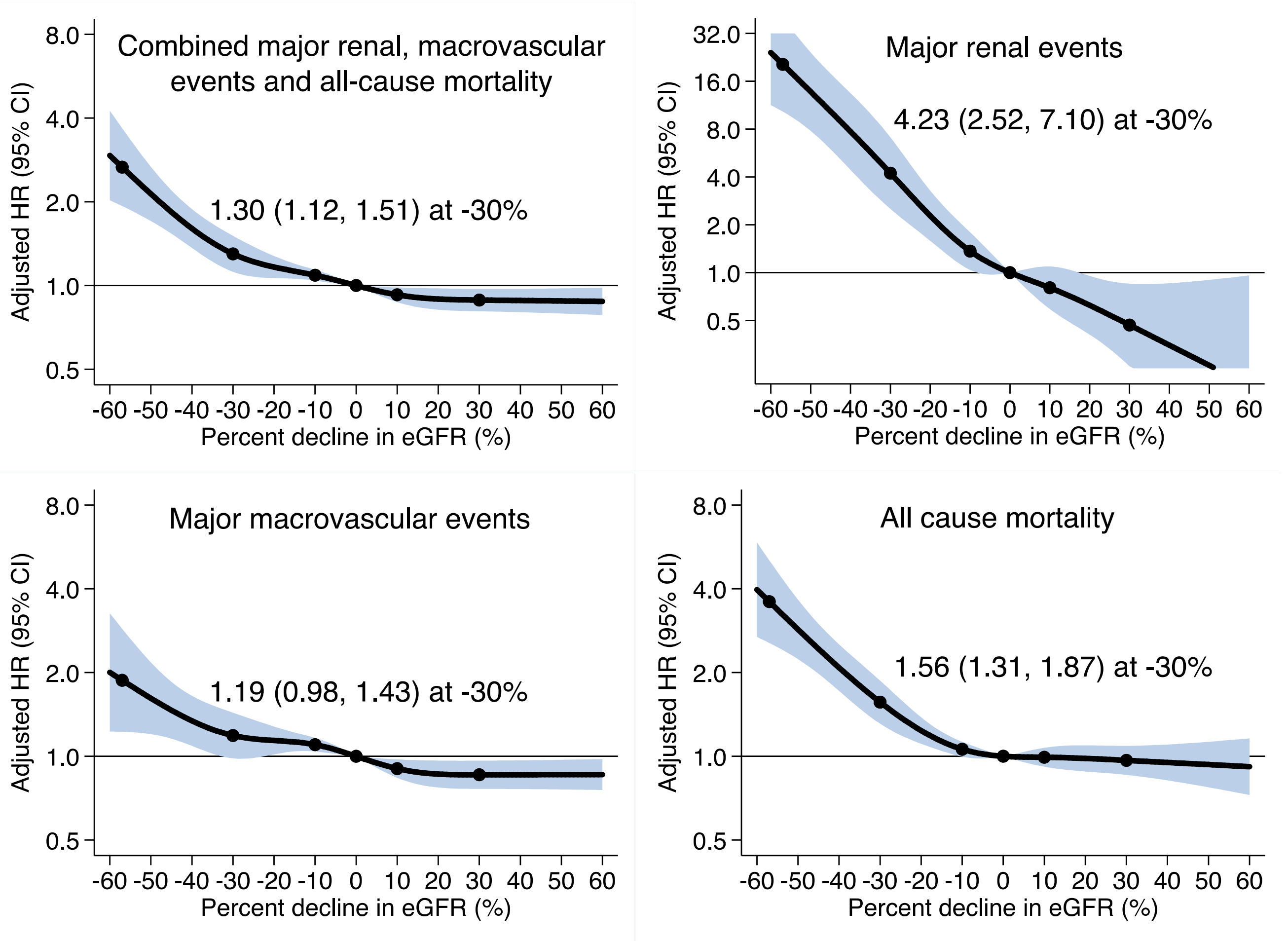

Values were trimmed at a percent change of <-60% and >60%. Knots were placed at -57, -30, -10, 10 and 30%, using 0% as the reference point. Covariates: registration values of age, sex, region of residence, duration of diabetes, log-transformed UACR, systolic BP, diastolic BP, a history of macrovascular disease, smoking, drinking, treated hypertension, HbA_{1c}, HDL-cholesterol, LDL-cholesterol, log-transformed triacylglycerol and BMI, 4-month eGFR and randomised treatment allocation (BP and glucose treatment)
